# Bacteremia Antibiotic Length Actually Needed for Clinical Effectiveness (BALANCE): study protocol for a pilot randomized controlled trial

**DOI:** 10.1186/s13063-015-0688-z

**Published:** 2015-04-18

**Authors:** Nick Daneman, Asgar H Rishu, Wei Xiong, Sean M Bagshaw, Deborah J Cook, Peter Dodek, Richard Hall, Anand Kumar, Francois Lamontagne, Francois Lauzier, John C Marshall, Claudio M Martin, Lauralyn McIntyre, John Muscedere, Steven Reynolds, Henry T Stelfox, Robert A Fowler, on behalf of the Canadian Critical Care Trials Group

**Affiliations:** Division of Infectious Diseases & Clinical Epidemiology, Sunnybrook Health Sciences Centre, University of Toronto and Adjunct Scientist, Institute for Clinical Evaluative Sciences, Sunnybrook Health Sciences Centre, 2075 Bayview Ave, Toronto, Ontario M4N 3M5 Canada; Department of Critical Care Medicine, Sunnybrook Health Sciences Center, 2075 Bayview Ave, Toronto, ON M4N 3M5 Canada; Division of Critical Care Medicine, University of Alberta Edmonton, 2-124E 8440-112 ST NW, Edmonton, AB T6G 2B7 Canada; Division of Critical Care Medicine, Department of Medicine, McMaster University, 1280 Main Street West, Hamilton, ON L8S 4L8 Canada; Division of Critical Care Medicine and Center for Health Evaluation and Outcome Sciences, St Paul’s Hospital and University of B.C, 1081 Burrard Street, Vancouver, BC V6Z 1Y6 Canada; Division of Critical Care Medicine, Department of Anesthesiology, Dalhousie University and the Capital District, Health Authority, 5790 University Avenue, Halifax, NS B3H 1V7 Canada; Section of Critical Care Medicine, University of Manitoba, 710 Park Blvd South, Winnipeg, MB R3P 0X1 Canada; Centre de recherche Clinique Étienne-Le Bel, 2500 boul. de l’Université, Université de Sherbrooke, Sherbrooke, QC J1K 2R1 Canada; Centre de recherche FRQS du Centre hospitalier affilié universitaire de Québec, Axe Traumatologie - urgence - soins intensifs, Division de soins intensifs adultes, départements de médecine et d’anesthésiologie, Université Laval, 1401, 18e Rue, Québec, QC G1J 1Z4 Canada; Departments of Surgery and Critical Care Medicine, St. Michael’s Hospital, University of Toronto, 30 Bond Street, Toronto, ON M5B 1W8 Canada; Department of Medicine, London Health Sciences Centre, University of Western Ontario, 800 Commissioners Rd. E, London, ON N6A 4G5 Canada; Division of Critical Care, Department of Medicine, The Ottawa Hospital, 501 Smyth Road, Ottawa, ON K1H 8L6 Canada; Department of Medicine, Kingston General Hospital, Queen’s University, 76 Stuart Street, Kingston, ON K7L 2V7 Canada; Department of Medicine, Royal Columbian Hospital, University of British Columbia, 260 Sherbrook Street, New Westminster, Vancouver, BC V3L 3M2 Canada; Department of Critical Care Medicine, Institute of Public Health, University of Calgary, 1403 29 Street NW, Calgary, AB T2N 2T9 Canada; Departments of Medicine and Critical Care Medicine, Sunnybrook Health Sciences Center, University of Toronto, 2075 Bayview Ave, Toronto, ON M4N 3M5 Canada

**Keywords:** intensive care, critically ill, bacteremia, bloodstream infection, antimicrobial, treatment duration, mortality, antimicrobial stewardship

## Abstract

**Background:**

Bacteremia is a leading cause of mortality and morbidity in critically ill adults. No previous randomized controlled trials have directly compared shorter versus longer durations of antimicrobial treatment in these patients.

**Methods/Design:**

This is a multicenter pilot randomized controlled trial in critically ill patients with bacteremia. Eligible patients will be adults with a positive blood culture with pathogenic bacteria identified while in the intensive care unit. Eligible, consented patients will be randomized to either 7 days or 14 days of adequate antimicrobial treatment for the causative pathogen(s) detected on blood cultures. The diversity of pathogens and treatment regimens precludes blinding of patient and clinicians, but allocation concealment will be extended to day 7 and outcome adjudicators will be blinded. The primary outcome for the main trial will be 90-day mortality. The primary outcome for the pilot trial is feasibility defined by (i) rate of recruitment exceeding 1 patient per site per month and (ii) adherence to treatment duration protocol ≥ 90%. Secondary outcomes include intensive care unit, hospital and 90-day mortality rates, relapse rates of bacteremia, antibiotic-related side effects and adverse events, rates of *Clostridium difficile* infection, rates of secondary infection or colonization with antimicrobial resistant organisms, ICU and hospital lengths of stay, mechanical ventilation and vasopressor duration in intensive care unit, and procalcitonin levels on the day of randomization, and day 7, 10 and 14 after the index blood culture.

**Discussion:**

The BALANCE pilot trial will inform the design and execution of the subsequent BALANCE main trial, which will evaluate shorter versus longer duration treatment for bacteremia in critically ill patients, and thereby provide an evidence basis for treatment duration decisions for these infections.

**Trial registration:**

The Pilot Trial was registered on 26 September 2014. Trial registration number: NCT02261506.

**Electronic supplementary material:**

The online version of this article (doi:10.1186/s13063-015-0688-z) contains supplementary material, which is available to authorized users.

## Background

Bacteremia is a common and serious problem affecting 15% of critically ill patients and resulting in a threefold higher mortality [1,2]. Among survivors, bacteremia is associated with a 2 to 7 day prolongation of intensive care unit (ICU) length of stay, a 2 to 3 week prolongation of hospital stay, and $25,000 to $40,000 in attributable excess costs [3,4].

The ICU is the location of greatest antimicrobial use in most hospitals; however, audits have revealed that 30 to 50% of antibiotic use in the ICU is unnecessary or inappropriate [5-7] and leads to avoidable drug side effects. Antibiotics are among the most common cause of serious adverse drug events [8], which occur in up to 5 to 10% of inpatient recipients [9]. Excessive durations of antibiotic therapy are the greatest contributor to inappropriate antibiotic use in acute care hospitals, long-term care facilities, and ambulatory care [6,10-12]. Discontinuing antibiotics after achieving clinical cure can potentially reduce the burden of adverse events (allergy and organ toxicity), *Clostridium difficile* infections, and additional costs and morbidity related to selection of antimicrobial resistant pathogens [13,14] Antibiotic resistance rates are rising among pathogens in ICUs [15] at the same time as the drug discovery pipeline is diminishing with very few new antimicrobial agents under development [16,17].

Meta-analysis of randomized controlled trials has demonstrated that shorter duration antibiotic therapy is as effective as longer duration antibiotic therapy for a range of mild to moderate infections [18]. Even in critically ill patients with ventilator-associated pneumonia, mortality rates and relapse rates were non-inferior among the 402 patients (of which only 7% were bacteremic) randomized to receive shorter (8 day) versus longer (15 day) courses of antibiotics [19]. However, similar high-grade evidence is lacking for the treatment of patients with bloodstream infections [1,20,21]. Specific guidelines for treatment durations exist for a variety of infections in ICU, including pneumonia [22,23], intra-abdominal infection [24], catheter-related bloodstream infection [25], pyelonephritis [26], and skin and soft tissue infection [27] but no guidelines exist for the optimal duration of treatment for bacteremic patients.

We have performed a systematic review of the existing literature [21], a national practice survey of Infectious Diseases (ID) and critical care physicians [28], a single center observational study [29], and a multicenter observational study [Daneman N, Rishu AH, Xiong W, Bagshaw SM, Dodek P, Hall R, et al: Antibiotic Treatment Durations Among Canadian Critically Ill Patients with Bacteremia, forthcoming]. We have identified gaps in current evidence, documented extensive practice variation, and confirmed equipoise for a randomized controlled trial comparing shorter (7 days) versus longer (14 days) antibiotic treatment durations for bloodstream infections.

The primary aim of the Bacteremia Antibiotic Length Actually Needed for Clinical Effectiveness (BALANCE) main randomized controlled trial will be to determine whether 7 days (as compared to 14 days) of adequate antibiotic treatment is associated with non-inferior survival for critically ill patients with bacteremia. The aim of this BALANCE pilot randomized controlled trial is to determine whether recruitment and protocol adherence rates will be sufficient for the main trial to be feasible.

## Methods/Design

### Design

We will conduct a multicenter randomized concealed allocation trial of shorter duration (7 days) versus longer duration (14 days) antibiotic treatment for critically ill patients with bacteremia admitted to ICU.

### Setting

To maximize efficiency, the BALANCE pilot trial will aim to involve a geographically and clinically diverse spectrum of ICUs across Canada. The study will be initiated in October 2014 at Sunnybrook Health Sciences Centre (SHSC) in Toronto, Ontario, and then rolled out to additional sites, starting with Kingston General Hospital in Kingston, Ontario. These two sites will be sufficient to accrue the intended pilot sample size of 115 patients, but we aim to roll out to 13 additional sites, if sufficient funding is acquired, to improve external generalizability of the pilot trial findings. See Additional file 1 for participating pilot sites.

### Population

All patients aged ≥18 years will be considered for enrolment in this study if they meet all the inclusion and no exclusion criteria.

#### Inclusion criteria

Patient is in the ICU at the time a blood culture result is reported as positive with a pathogenic bacterium.

#### Exclusion criteria

1. Patient has severe immune system compromise, as defined by: absolute neutrophil count <0.5×10^9^/L; or is receiving immunosuppressive treatment for solid organ or bone marrow or stem cell transplant.
2. Patient has a prosthetic valve or synthetic endovascular graft.
3. Patient has a suspected or documented syndrome with well-defined requirement for prolonged treatment (infective endocarditis, osteomylitis/septic arthritis, undrainable/undrained abscess, or unremovable/unremoved prosthetic-associated infection).
4. Patient has a single positive blood culture with a common contaminant organism according to the Clinical Laboratory & Standards Institute (CLSI) (coagulase negative staphylococci, *Bacillus spp., Corynebacterium spp., Propionobacterium spp., Aerococcus spp., Micrococcus spp*) [30].
5. Patient has a positive blood culture with *Staphylococcus aureus* [31].
6. Patient has a positive blood culture with *Candida spp.* or other fungal species as the only potential pathogen.
7. Patient already or previously enrolled in the trial.

### Eligible, nonrandomized patients

We will maintain a log of all patients who were eligible but not randomized due to one of the following reasons:

1. Patient or substitute decision maker (SDM) declined consent, specifying reason;
2. Patient unable to give consent and SDM not available;
3. ICU physician declined consent, specifying reason; [32]
4. Consent not obtained due to other reason, specifying reason.

### Ethics and informed consent

The study protocol has been approved by the Sunnybrook Health Sciences Research Ethics Board and the Queen’s University Health Sciences and Affiliated Teaching Hospitals Research Ethics Board (for Kingston General Hospital), and will be sought at other participating sites. After seeking permission from the treating team, the research coordinator/site primary investigator will approach eligible patients (or their substitute decision-makers) as soon as their blood cultures are positive to obtain informed consent (see Additional file 2). Consent can be delayed at maximum to the 7th day of adequate antibiotic treatment. Critically ill patients are frequently unable to provide initial consent due to altered level of consciousness or understanding. Hence, the Canadian Critical Care Trials Group (CCCTG) has standard operating procedures to seek assistance from substitute-decision makers on behalf of patients. This process has been found feasible and acceptable to patients, decision-makers, and research ethics boards across Canada and has been successfully employed among dozens of CCCTG RCTs [32-35]. We will use this enhanced approach to consent, employing 13 previously described strategies distributed over three phases: preparation for the consent encounter, the consent encounter, and as follow-up to the consent encounter [34].

### Randomization and allocation concealment

Randomization will occur as soon as the consent is obtained. The web-based randomization system for BALANCE will be created using RANDOMIZE.NET (http://www.randomize.net/), and will employ variable block sizes, stratified by ICU site. After the full susceptibility results become available, the site research coordinator along with site co-investigators will determine the date for day 7 unblinding, taking into account the number of days that the patient has already received adequate antibiotics after the blood culture collection date. At day 7 (date entered by the research coordinator), another email will be sent with the unblinded treatment assignment for the patient. At this time the unblinded treatment for that patient will be displayed in the reports available to the site research coordinator. If a patient is randomized to the shorter (7 day) treatment arm, the treating team will be informed to stop the antibiotics at the completion of 7 days of antibiotics appropriate for the causative pathogen; if the patient is randomized to the longer (14 day) arm the team will be instructed to continue the antibiotic until that date.

### Trial interventions

**Figure 1:**
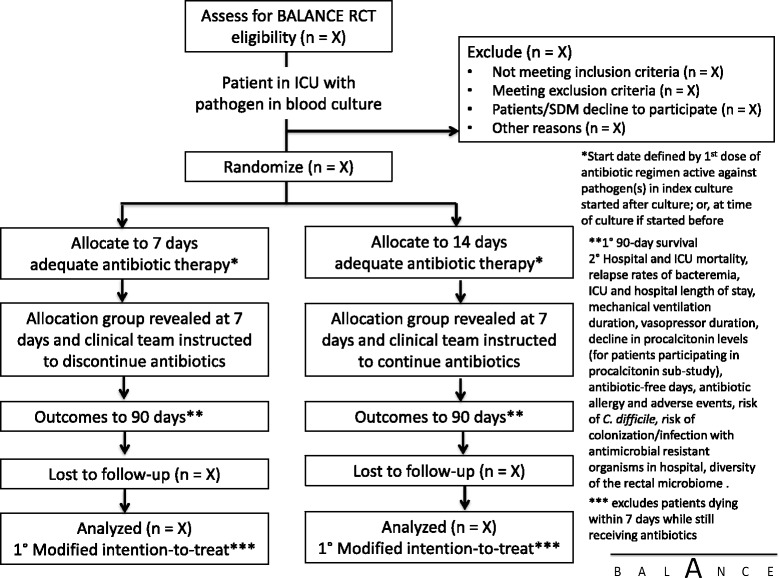
BALANCE pilot RCT intervention flow diagram.

We will randomize patients to receive a shorter duration of adequate antimicrobial therapy (7 days) versus a longer duration (14 days). Adequate antimicrobial treatment will be defined as a regimen with *in vitro* activity against the organism(s) responsible for the bloodstream infection; the duration of adequate treatment will be determined as the cumulative number of calendar days for which adequate treatment is delivered beyond the date of collection of the index blood culture specimen- the clock will start from initiation of the first adequate treatment dose after the blood culture has been drawn. The selection of specific antimicrobial agents, doses and route of delivery will initially be at the discretion of the treating clinical team. Most commonly the patient will already be receiving some empiric antibiotic treatment for suspected infection prior to the blood culture being documented as positive; the study protocol will not expose patients to potential antibiotic treatment delays. As soon as blood culture results are available (preliminary Gram staining), the research coordinator, in consultation with the site investigator and site infectious diseases co-investigator if necessary, will review the initial antibiotic choice to ensure adequate empiric coverage for the potential culprit organism(s). As blood culture results are finalized (speciation and sensitivity determination), they will re-review the antibiotic choice to ensure that the spectrum of activity is adequate. If the spectrum is inadequate, this will be communicated to the treating physicians. To avoid differentially influencing antibiotic choices and clinical decision-making, the randomization assignment will not be communicated to the study research coordinator, study critical care or infectious diseases investigators or clinicians until the end of day 7. The ICU research coordinator at each site will visit daily to ensure protocol adherence (antibiotics are stopped at the pre-specified date (end of the 7th or 14th day) and not earlier (Figure 1).

**Figure 2:**
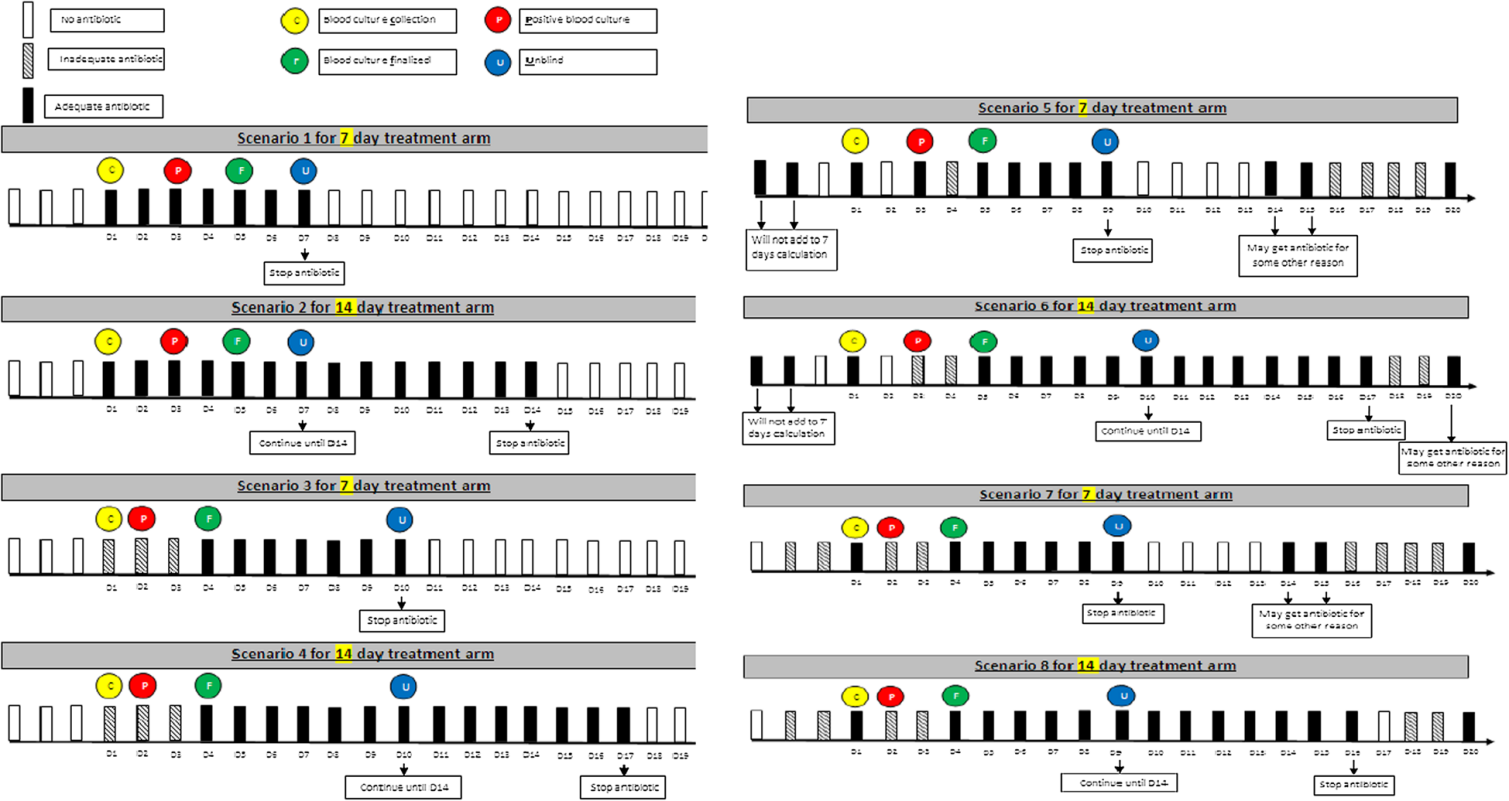
Determination of day 7 of antibiotic treatment. Multiple scenarios are provided to determine how day 7 of antibiotic treatment will be determined based on days of receipt of adequate antibiotic treatment after collection of the positive blood culture. Adequacy of treatment will only be measurable after the culture and susceptibility result has been finalized.

Calculating day 7 of adequate antibiotics is a complex procedure; there are multiple potential scenarios of adequate and inadequate antibiotic treatment days prior to the culture results being finalized. Figure 2 demonstrates some of the possible scenarios, to illustrate calculation of day 7 from the positive blood culture collection date. The day 7 unblinding date will be calculated as the cumulative number of calendar days the patient receives adequate antibiotics after the positive blood culture collection date. Adequacy of antibiotics will only be definable after the final susceptibility report is available. If a patient received inadequate antibiotics after the positive blood culture collection and before the culture is finalized (Figure 2, scenario 3), day 7 date will be calculated from the date adequate antibiotics were started, which should be immediately after the culture is finalized. If a patient received adequate antibiotics for one day after positive blood culture collection and then was switched to inadequate antibiotics until the culture was finalized (Figure 2, scenario 7), then this one day of adequate antibiotic after positive culture collection will still be counted in the calculation of the day 7 date. Once the culture is finalized, adequate antibiotic treatment should be started/continued without any break until the completion of the assigned treatment duration based on study arm. Discontinuation or missed treatment before the completion of the assigned treatment duration will be considered to be a protocol violation.

### Blood samples for procalcitonin

Blood samples will be drawn on the randomization day and at days 7, 10 and 14 from the index blood culture collection to measure procalcitonin (PCT) levels. The PCT levels will be batched and measured at the end of the study for the sub-study assessing the association between PCT and clinical outcomes among patients receiving 7 versus 14 days of treatment. The results will not be made available to the treating team, because this could unduly influence clinical practice and protocol adherence, and is ethical because none of the participating sites are currently using PCT as part of routine clinical practice. Following study completion, we will compare PCT area-under-the-curve (AUC) and day 14 PCT levels among patients receiving 7 versus 14 days of antibiotics. We will also confirm whether 7 days of antibiotics is non-inferior to 14 days of antibiotics for bacteremia, in subgroups with both normal and abnormal PCT levels on day 7.

### Protecting against sources of bias

#### Selection bias

Selection bias (such as bias-by-indication or survival bias) will be minimized through rigorous concealed randomization procedures. Although placebo controls have been used in some RCTs of antibiotic treatment duration, such as studies examining treatment duration for cellulitis [36], pyelonephritis [37,38], and community-acquired pneumonia [39-42], they are not appropriate for bacteremia treatment in the ICU. It will not be practical to provide placebos for each of the many antimicrobials commonly used alone or in combination to treat bacteremia due to varied infectious syndromes and pathogens; in our multicenter retrospective study more than 100 different species were identified in enrolment blood cultures, and more than 60 different individual antimicrobials were administered [43]. Even if it were feasible to generate this many placebos, patients in critical care may develop secondary sources of nosocomial infection, and clinicians will not agree to be unaware of whether their patients are already receiving active antimicrobial treatments versus placebo. The successful, landmark *PneumoA* trial of shorter versus longer duration treatment for ventilator associated pneumonia, did not employ placebos [19].

### Outcome misclassification bias

We have selected objective outcome measures, and we will use central adjudication committees blinded to treatment allocation for subjective outcomes including secondary infection or colonization with antibiotic resistant organisms [44,45].

### Publication bias

We will prevent publication bias by registering both the BALANCE Pilot Trial and the main BALANCE Trial, and through this publication of the trial protocol.

### Withdrawal from study

All consented patients will be followed regardless of adherence to the trial protocol. If a patient is withdrawn from the study prematurely, a withdrawal form will be completed. Data will be collected under the informed consent up to the point of a consent withdrawal. If permitted by the patient or substitute-decision maker, data collection will continue for withdrawn patients. Primary analyses will be carried out on the basis of *intention-to-treat* principle, safety outcomes will be assessed using per-protocol analyses. Anticipated reasons for withdrawal include patient not meeting inclusion criteria or relevant exclusion criteria present prior to randomization, consent withdrawn by patient or substitute-decision maker, patient’s physician believes patient should be withdrawn from the study, inadvertent duplicate randomization. Detailed rationales for withdrawal will be recorded.

### Protocol violation

A protocol violation form will be completed in case of the following violations:

1. Adequate antibiotic treatment stopped by treating physician before RCT-dictated antibiotic stop date
2. Adequate antibiotic treatment continued by treating physician after RCT-dictated antibiotic stop date

We will also document the reason for protocol violation, responsible person for protocol violation (for example, attending physician, trainee physician, nurse, pharmacist, *etcetera*) and if there were any clinical explanations that might have resulted in the protocol violation.

### Frequency and duration of follow-up

The antibiotic treatment durations may extend beyond ICU discharge for some patients, and beyond hospital discharge for a minority. Our retrospective observational study showed that 27% of patients were discharged from ICU to hospital wards on or before day 7 of adequate antibiotic treatment and 4% were discharged from hospital before day 7. Patients will be reviewed daily in ICU, at hospital discharge and at 90 days post-randomization. If a patient is discharged from hospital prior to 90 days post-randomization, the research coordinator will contact the patient (or substitute decision-maker as appropriate) by telephone to determine their disposition and vital status.

### Study drug related daily data

The research coordinator will assess the patient daily (each morning) for 14 days after randomization to ensure the clinical team adheres to the study treatment duration protocol. If the clinical team proposes stopping antibiotics prior to the assigned stop date, the research coordinator and/or site investigator will follow-up with the clinical team to continue appropriate antibiotics; if the team extends antibiotics beyond the assigned stop date the research coordinator will suggest discontinuing the antibiotics.

### Primary and secondary outcome measures

The primary outcome for the main BALANCE trial is 90-day mortality and the primary outcome for the pilot trial is feasibility as defined by: (a) adherence to treatment duration protocol (proportion of treatment courses); and (b) rate of recruitment (patients per site, per month). We will consider the main trial to be feasible and worthy of embarking on a larger mortality-powered non-inferiority RCT if 90% of antibiotic treatment courses are within 7 ± 2 days in the shorter duration treatment arm or 14 ± 2 days in the longer duration treatment arm; and, if we achieve recruitment rates of at least 1 patient per 4 weeks, on average, per participating site. Secondary outcomes include ICU, hospital and 90-day mortality rates, relapse rates of bacteremia, antibiotic allergy and adverse events, rates of *Clostridium difficile* infection in hospital, rates of secondary infection or colonization with antimicrobial resistant organisms, ICU and hospital lengths of stay, mechanical ventilation duration, vasopressor duration in ICU and decline in procalcitonin levels.

### Statistical analysis

#### Sample size

To estimate our expected adherence rate of 90% within a margin of error of ± 5% (that is, actual adherence 85 to 95%), with 95% confidence, we plan to enroll 115 patients. At our expected recruitment rate of 1 patient/site month across 15 sites, we will need approximately 10 to 24 months to recruit these 115 patients given variability in site trial start-up steps (REB approval, data sharing agreements and contracts). By the time we have recruited 115 patients, we will know with 95% confidence that this number of sites would be able to recruit 95 to 138 patients over the same time interval in a future trial; this data will then be used to estimate the number of centers needed for the main trial (see Additional file 1 for detailed sample size calculation).

### Loss to follow-up

We anticipate negligible loss of patients to follow-up. The Canadian Critical Care Trials Group (www.ccctg.ca), who endorsed this trial, has achieved virtually 100% follow-up to hospital discharge over all of its landmark RCTs [46-48]. Although we will be following survivors to ascertain 90-day mortality and relapse rates, we also expect close to 100% follow-up based on our previous CCCTG experience.

### Analysis of primary outcomes

The primary outcomes will be analyzed as follows:

(i). *Rate of recruitment*: We will consider the main trial to be feasible if we achieve recruitment rates of at least 1 patient per month per participating site. We will report recruitment rates with 95% confidence intervals.
(ii). *Adherence to treatment duration protocol*: We will consider the main trial to be feasible if 90% of antibiotic treatment courses are 7 ± 2 days in the shorter duration treatment arm, and at least 90% of antibiotic treatment courses are 14 ± 2 days in the longer duration treatment arm. We will report proportion of adherence with 95% confidence intervals.

### Analysis of secondary outcomes

Mortality rates (at ICU discharge, hospital discharge and 90 days) will be measured as the proportion of patients alive or dead at these time points. We hypothesize that mortality rates will be non-inferior with 7 days of treatment; the main BALANCE trial will be powered to test this hypothesis. Continuous secondary outcomes, including lengths of stay in ICU and hospital, and durations of ventilation and vasopressor use, will be compared by Wilcoxon test. We will compare the ‘difference in differences’ between day 7 to 14 procalcitonin levels in the two treatment groups.

### Frequency of analyses

The BALANCE Pilot Trial will be conducted and reported according to recommendations by the Consolidated Standards of Reporting Trials (CONSORT), including analyzing patients in the groups to which they were originally assigned (intention-to-treat) and explicit procedures for handling of any missing data [49].

Analyses will be conducted at the completion of this pilot, given that there is no role for early termination [50]. However, we will monitor recruitment and adherence rates weekly, and examine barriers as needed, working with participating centers to provide screening and consenting tips from prior CIHR-funded ICU trials [34]. Capturing a minimal dataset of patient, pathogen and bacteremia source characteristics among eligible nonenrolled patients will facilitate this, and comparisons with eligible patients will help to identify selection bias [49].

### Subgroup analyses

To inform and possibly refine the main BALANCE Trial design, we will perform four exploratory subgroup analyses. The main subgroup analysis will be based on the underlying infectious syndrome causing bacteremia (vascular catheter-related, pneumonia, pyelonephritis, intra-abdominal, skin and soft tissue, other identified source, or unknown source). We will also perform subgroup analyses based on illness severity (APACHE II score of ≥25 versus <25), community- versus hospital-acquisition, and vasopressor use on day of randomization. If recruitment rates or protocol adherence are particularly low for any subgroups, in consultation with the Steering Committee and CCCTG, we will consider modifying definitions or procedures appropriately. For safety analyses, we will perform a per-protocol analysis by restricting the analysis to the participants who adhered perfectly to the protocol in terms of the eligibility, interventions, and outcome assessment.

### Steering committee

The Steering Committee is responsible for development and oversight of the BALANCE Pilot and full RCT procedures and operations, funding applications, recruitment rates, missing data rates, in addition to advising the principal applicants on responses to questions from the data safety and monitoring committee or other stakeholders, and eventual interpretation and compilation of study results into reports, scholarly manuscripts and knowledge translation and exchange activities.

The steering committee will meet in-person thrice yearly at CCCTG meetings (November, Ontario; January, Alberta; June, Eastern Canada) and by teleconference as needed between meetings.

### Data Safety and Monitoring Committee (DSMC)

After broad discussion and engagement of the CCCTG it was determined that a DSMC would not be required for this pilot study, given the short duration of enrolment, and that it is underpowered to examine mortality. A DSMC will be instituted for the subsequent main BALANCE trial [51]. DSMC composition will follow accepted norms of content expertise (critical care, infectious diseases, methodology, and biostatistics) and independence from the investigators and steering committee.

## Discussion

### Rationale for studying bacteremia

Some might argue that duration of antibiotic treatment should be driven by the underlying infectious focus, rather than the presence or absence of bacteremia. However, in our national practice survey the distributions of treatment duration recommendations were virtually identical for scenarios of bacteremic pneumonia, bacteremic pyelonephritis, catheter-related bloodstream infection, bacteremic intra-abdominal infection, and bacteremic skin and soft tissue infection - highlighting that bacteremia is a very influential syndromic aspect and the appropriate focus for our research program [28]. The advantages of studying bacteremia as a clinical entity versus other associated syndromes outweigh any potential disadvantages. In contrast to syndromic diagnoses (ventilator-associated pneumonia for example), all patients with bacteremia have a positive sterile site culture result (by definition). Therefore, all bacteremic patients (with noncontaminant species) have true infection, whereas the presence or absence of pneumonia is much harder to define because cultures may represent colonization rather than infection, and even multiple adjudications of case definitions provide only moderate agreement, particularly in patients on mechanical ventilators [52,53]. Given that bacteremia is defined by the positive blood culture result, all study patients will have an identified pathogen, in contrast to syndromic infections, which are often treated empirically without a defined etiology. A corollary is that antibiotic susceptibility test results are available for all bacteremic patients, so it will be clear whether or not patients randomized to shorter versus longer duration antibiotic treatment are receiving an effective antibiotic. The bacteremic subgroups of patients with pneumonia, pyelonephritis, intra-abdominal infection, and soft tissue infection, generally have more severe and complicated courses than non-bacteremic infections. Therefore, if shorter course therapy is demonstrated to be effective for bacteremic patients, the results can be more easily generalized to non-bacteremic patients than vice versa. Finally, pre-specified subgroup analyses in the main BALANCE RCT can examine the impact of treatment duration within each specific syndrome.

### Rationale for studying fixed duration therapy versus individualized durations based on clinical or biomarker based stopping rules

Ideally antibiotic treatment duration should be individualized, and patients should be treated until their infection has been cured (and likely no longer) [24]. Unfortunately, a randomized controlled trial based on a clinical stopping rule is not feasible in ICU patients, because there are no specific markers of persistent infection during critical illness. The difficulty in diagnosing infection in ICU and monitoring clinical response to treatment, has generated considerable interest in the use of novel biomarkers to guide antibiotic treatment duration [42,54]. One biomarker, procalcitonin, has been used successfully to reduce average treatment durations in sepsis [42]. However, only a minority of these patients were bacteremic. Moreover, more than half of patients randomized to the procalcitonin group, were not given algorithm-guided treatment because the attending physician believed a biomarker-informed duration was inappropriate [54].

Instead, we favor a randomized trial of fixed shorter versus longer duration antibiotic therapy, guided by our preliminary studies, as the most easily transferrable result to immediately inform clinical practice. This approach has been successful in more than two dozen randomized controlled trials of infectious diseases that are potentially complicated by bacteremia [21]. Most notably, a trial in ventilator-associated pneumonia has altered the standard of care for this infection to shorter duration therapy (8 days) [19]. However, appreciating the future promise of individual patient focused biomarkers to further nuance treatment decisions, we will measure procalcitonin levels and trajectory in both treatment arms to see if it could provide incremental prognostic value [49].

## Trial status

The study protocol is approved by the Sunnybrook Health Sciences Centre and Kingston General Hospital REBs. Funding has been obtained for this pilot RCT from the Ministry of Health and Long-Term Care Academic Health Sciences Alternative Funding Plan Innovation Fund Award (Ontario, Canada). The case report forms (paper and electronic) have been finalized. Training will be done for all the participating sites through webinar before starting recruitment. The study is registered with randomize.net and randomization of test patients has been successfully achieved. The trial is expected to begin enrolling patients in October 2014. Recruitment of patients will start at Sunnybrook Health Sciences Centre (coordinating center) and then roll out successively to the other sites after obtaining REB approval at the individual sites, and finalizing data sharing agreements. With the planned recruitment, we expect to complete this pilot trial in the summer of 2016 and then enroll further Canadian and International centers for the main BALANCE RCT. If the pilot RCT does not identify a need for any substantive protocol changes, this will be an internal pilot trial, and patients will be rolled into the main trial.

## Additional files

Additional file 1:
**Participating Sites.** List of potential participating sites for the Bacteremia Antibiotic Length Actually Needed for Clinical Effectiveness (BALANCE) pilot randomized controlled trial. Additional file 2:
**Informed Consent Form.** Informed consent form for the Bacteremia Antibiotic Length Actually Needed for Clinical Effectiveness (BALANCE) pilot randomized controlled trial.

## Abbreviations

APACHE: Acute physiology and chronic health evaluation
CCCTG: Canadian critical care trials group
CIHR: Canadian institutes of health research
CLSI: Clinical laboratory & standards institute
CONSORT: Consolidated standards of reporting trials
DSMC: Data safety and monitoring committee
ICU: Intensive care unit
ID: Infectious diseases
RCT: Randomized control trial
SDM: Substitute decision maker
