# Supplementary material for: Bacteremia Antibiotic Length Actually Needed for Clinical Effectiveness (BALANCE): study protocol for a pilot randomized controlled trial
Source: Trials. 2015 Apr 18;16:173. doi: 10.1186/s13063-015-0688-z (PMC4407544; doi:10.1186/s13063-015-0688-z)
Supplement: Additional file 1: — Participating Sites. List of potential participating sites for the Bacteremia Antibiotic Length Actually Needed for Clinical Effectiveness (BALANCE) pilot randomized controlled trial. [file 13063_2015_688_MOESM1_ESM.pdf]

Sample size calculation

Non-Inferiority Margin	Baseline mortality†	Alpha*	Power	Sample Size Per Arm
-1%	27%	0.025	90%	41421
-1%	27%	0.025	80%	30941
-1%	27%	0.050	90%	33759
-1%	27%	0.050	80%	24372
-2%	27%	0.025	90%	10356
-2%	27%	0.025	80%	7736
-2%	27%	0.050	90%	8440
-2%	27%	0.050	80%	6094
-3%	27%	0.025	90%	4603
-3%	27%	0.025	80%	3439
-3%	27%	0.050	90%	3752
-3%	27%	0.050	80%	2709
-4%	27%	0.025	90%	2589
**-4%**	**27%**	**0.025**	**80%**	**1934**
-4%	27%	0.050	90%	2111
-4%	27%	0.050	80%	1524
-5%	27%	0.025	90%	1658
-5%	27%	0.025	80%	1238
-5%	27%	0.050	90%	1351
-5%	27%	0.050	80%	975

Participating sites

Site Investigator	Infectious Disease Specialist	Site Coordinator	Hospital
Nick Daneman, MD**<br>Rob Fowler, MD**	Nick Daneman, MD	Asgar Rishu	Sunnybrook Health Sciences, Toronto, ON
Sean Bagshaw, MD	Wendy Sligl, MD	Samantha Taylor	University of Alberta Hospital, Edmonton, AB
Peter Dodek, MD	Victor Leung, MD	Betty Jean Ashley	St. Paul's Hospital, Vancouver, BC
Rick Hall, MD	Lynn Johnston, MD	Lisa Julien	Queen Elizabeth II Halifax, NS
Anand Kumar, MD	Anand Kumar, MD	Wendy Janz	St. Boniface Hospital, Winnipeg, MB
François Lamontagne, MD	Alex Carignan, MD	Chantal Langevin	Université de Sherbrooke, Sherbrooke, QC
François Lauzier, MD	Julie Bestman-Smith, MD	France-Emilie Roy	Centre hospitalier affilié universitaire de Québec, Québec, QC
John Marshall, MD	Linda Taggert, MD	Orla Smith	St. Michael's Hospital, Toronto, ON
Claudio Martin, MD	Sameer El Sayed, MD	Eileen Campbell	London Health Sciences Centre, London, ON
Lauralyn McIntyre, MD	Baldwin Toye, MD	Irene Watpool	The Ottawa Hospital, Ottawa, ON
John Muscedere, MD	To Be Determined, MD	Susan Fleury	Kingston General Hospital, Kingston, ON
Steven Reynolds, MD	Steve Reynolds, MD	Suzette Willems	Royal Columbian Hospital, Vancouver, BC
Tom Stelfox, MD	John Conly, MD	Dan Lane	Foothills Hospital, Calgary, AB
