# Supplementary material for: Bacteremia Antibiotic Length Actually Needed for Clinical Effectiveness (BALANCE): study protocol for a pilot randomized controlled trial
Source: Trials. 2015 Apr 18;16:173. doi: 10.1186/s13063-015-0688-z (PMC4407544; doi:10.1186/s13063-015-0688-z)
Supplement: Additional file 2: — Informed Consent Form. Informed consent form for the Bacteremia Antibiotic Length Actually Needed for Clinical Effectiveness (BALANCE) pilot randomized controlled trial. [file 13063_2015_688_MOESM2_ESM.pdf]

Sunnybrook Health Sciences Centre
2075 Bayview Avenue,
Toronto, ON Canada M4N 3M5
t: 416.480.6100
www.sunnybrook.ca

INFORMED CONSENT TO PARTICIPATE IN A RESEARCH STUDY

Study Title: Bacteremia **A**ntibiotic **L**ength **A**ctually **N**eeded for **C**linical **E**ffectiveness (**BALANCE**):

A Pilot Randomized Controlled Clinical Trial

PRINCIPAL INVESTIGATORS:
Nick Daneman, MD.
Dept of Medicine Division of Infectious Diseases
Sunnybrook Health Sciences Centre
University of Toronto
2075 Bayview Ave. Room D478
Toronto, Ontario, Canada, M4N 3M5
Contact: 416-480-6100 x2791
Pager: 7717
AND
Rob Fowler, MD, FRCPC.
Dept of Medicine and Critical Care Medicine
Sunnybrook Health Sciences Centre
Associate Professor, University of Toronto
2075 Bayview Ave. Room G106
Toronto, Ontario, Canada, M4N 3M5
Contact: 416-480-6100-7471

PROJECT MANAGER:
Asgar Rishu, MBBS
Sunnybrook Hospital
Trauma, Emergency and Critical Care Program
2075 Bayview Ave. Room K3W-17
Toronto, ON, Canada, M4N 3M5
Phone: 416-480-6100-ext 88153

Sponsor: Ministry of Health and Long-Term Care Academic Health Sciences Alternative Funding Plan Innovation Fund Award and Sunnybrook Health Sciences Centre.

Sunnybrook Health Sciences Centre
2075 Bayview Avenue,
Toronto, ON Canada M4N 3M5
t: 416.480.6100
www.sunnybrook.ca

This consent is directed to the patient, but, in the event that the patient is unable to give informed consent on his/her behalf, a next-of-kin or legal representative may provide consent on the patient's behalf.

INFORMED CONSENT

You are being asked to consider participating in a research study. A research study is a way of gathering information on a treatment, procedure or medical device or to answer a question about something that is not well understood.

This form explains the purpose of this research study, provides information about the study, the tests and procedures involved, possible risks and benefits, and the rights of participants. Please read this form carefully and ask any questions you may have. You may have this form and all information concerning the study explained to you. If you wish, someone may be available to verbally translate this form into your preferred language.

Please ask the study staff or one of the investigator(s) to clarify anything you do not understand or would like to know more about. Make sure all your questions are answered to your satisfaction before deciding whether to participate in this research study.
Participating in this study is your choice (voluntary). You have the right to choose not to participate, or to stop participating in this study at any time.

INTRODUCTION

You are being asked to consider participating in this study because you/your family member has developed blood stream infection. Blood stream infection means presence of bacteria in the blood which causes illness and is common in patients during ICU stay. In some people it is the cause of ICU stay, and in other people it develops during ICU stay. Such infections require urgent treatment with antibiotics. Limited literature is available to guide physicians about how long to treat these patients. Long durations of treatment (14 days) may result in unnecessary side effects and antibiotic resistance. Recent data from some research studies has suggested that shorter duration of antibiotic treatment ($\leq$7 days) is as effective as longer duration of treatment for a lot of infections due to bacteria but this has not been confirmed/tested in patients with blood stream infections.

WHY IS THIS STUDY BEING DONE?

The purpose of this study is to find the best possible duration of antibiotic treatment in patients with blood stream infections. There are no guidelines for physicians as to how long to treat patients with blood stream infection, and so there is no standard of care for treatment of

Sunnybrook Health Sciences Centre
2075 Bayview Avenue,
Toronto, ON Canada M4N 3M5
t: 416.480.6100
www.sunnybrook.ca

patients with blood stream infections. Physicians treat these patients based on their personnel experience and choice. There is wide variation in treatment duration; some patients are treated for a few days while others are treated for many days due to lack of evidence. The most common treatment choices by Canadian ICU doctors are 14, 10 and 7 days. Therefore, there is an urgent need to do a research study to determine the best antibiotic treatment duration in these patients. This research study is NOT testing a new drug. The antibiotics used in this study will all be standard medications routinely used in the ICU that are selected by the ICU team.

WHAT WILL HAPPEN DURING THIS STUDY?

As part of routine care, patients suspected to have infection will have their blood sample sent to Microbiology laboratory by the treating team to see if there is any infection and to find the bacteria causing it. These blood tests will be screened by the research team to see if the patient is eligible to be included in this study. After checking for all inclusion criteria and if you agree to have you/your family member participate and sign this informed consent form, you/your family member will be randomly assigned to one of the two groups: short course (7 days) or long course (14 days) of antibiotic treatment. The duration of treatment that you receive will be determined by chance. That is, there is a 50% chance of receiving 7 days and 50% chance of receiving 14 days of treatment.

The selection of antibiotics, doses and route will be initially done by the treating physician. As soon as preliminary blood test results are available, a pharmacist will consult with the infectious diseases physician and will review the antibiotics to make sure patient is receiving the right antibiotics for the bacteria causing their infection. After the blood test results are finalized, they will re-review the antibiotic to ensure that the patient is still getting the best possible antibiotic. If the antibiotic is not adequate, the treating team will be informed to change the antibiotic to adequate ones.

You will be followed by the research team in the ICU to collect some data like age, sex, hospital admission dates, severity of disease, reason for ICU admission and underlying disease etc. We will also collect data regarding signs and symptoms of any new infections, blood test results, any side effects, and status at the time of ICU and hospital discharge (alive or dead). If a patient is discharged from hospital within 90 days of blood stream infection, we will contact you by telephone to determine your/your family member’s status at the end of 90 days.

HOW MANY PEOPLE WILL TAKE PART IN THIS STUDY?

Sunnybrook Health Sciences Centre
2075 Bayview Avenue,
Toronto, ON Canada M4N 3M5
t: 416.480.6100
www.sunnybrook.ca

It is anticipated that about 115 patients will participate in this study at about 13 ICUs throughout Canada. About 30-40 people are expected to participate in this study at Sunnybrook Hospital. The length of this study for participants is up to a maximum of 14 days. The entire study is expected to take about 2 years to complete and the results should be known in 2-3 years.

WHAT ARE THE RESPONSIBILITIES OF STUDY PARTICIPANTS?

- To read and understand the consent form carefully and ask the research team any questions you may have.
- Sign the informed consent form.
- Provide the contact information to allow the study coordinator to contact you to find the status at day 90 if discharged from hospital.
- Take the study medication as instructed.
- Talk to the Principal Investigator (PI; the person in charge of the study) if you/your family member want to stop being part of the research study.
- Contact the PI and/or Sunnybrook Research Ethics Board with complaints or concerns about your participation in the study.
- Inform the PI immediately of any and all problems you/your family member may be having with the study medication.
- Keep a copy of the consent form for your records.

BLOOD SAMPLES

The blood sample(s) will be drawn on the day of inclusion in this study (day 0), 7, 10 and 14 to measure the procalcitonin levels, believed to be an indicator of bacterial infection. This test is not done routinely as a part of treatment and will have no effect on treatment choices. The collection of samples is optional for a sub-study directly related to the main research study but we would appreciate your consent for this test. You may decide not to have your blood sample collected and still participate in this study. If you don't want to have your blood sample collected, please use the check boxes on the signatory page. The blood sample is to be used for the current research study only and will not be used for any other test, research and will not be sold. To perform this test, 4 ml of blood will be obtained on each specified study day. The samples can be combined with the routine daily blood samples that are drawn to perform various blood tests during ICU stay and pose no major risk to you/your family member. We will store these samples in a safe and secure freezer in the Sunnybrook research laboratory till the study is completed in approximately 2 years. The blood samples will be discarded once it has

Sunnybrook Health Sciences Centre
2075 Bayview Avenue,
Toronto, ON Canada M4N 3M5
t: 416.480.6100
www.sunnybrook.ca

been used for the purposes described in this form. The results of the test will not be made available to the treating team. In case you want to know you results, please contact the investigators at the end of study. Any of your samples if needed to be sent outside of the hospital will have a code only and will not contain your name or address, or any information that directly identifies you. In case you/your family member is discharged form hospital before the due test date, you/your family member does not have to come back for the remaining tests.

WHAT ARE THE RISKS OR HARMS OF PARTICIPATING IN THIS STUDY?

You may experience some side effects from participating in this study. However, these side effects exist even if you do not participate in this research study because all patients with blood stream infections will be prescribed antibiotics for a variable duration of time. Some side effects are known and are listed below, but there may be other side effects that are not expected. If you/your family member decide to take part in this study, you should contact Dr. Nick Daneman (contact details are on top of page 1 of this document) about any side effects that you experience.

Some of the common known antibiotic side effects are: rash, diarrhoea, nausea/vomiting, headache, abdominal pain, hypersensitivity (allergic) reactions, renal (kidney) toxicity, ototoxicity (hearing loss), dizziness. In addition, short course (7 days) treatment duration can increase the risk of treatment failure or re-appearance of the bloodstream infection. Long course (14 days) treatment on the other hand may increase the chance of resistance to antibiotics, occurrence of new antibiotic-resistant infections, *Clostridium difficile* infection, and side effects like allergy, anaphylaxis, antibiotic related kidney injury, antibiotic related liver failure, and other antibiotic related organ toxicity. In case of any of these events, appropriate measures will be taken to prevent harm to the patient. There is also a possibility of pain, bruising, swelling or infection related to giving blood sample. These discomforts are minimal and brief. All of these side effects could occur for patients with bloodstream infections that are not enrolled in this trial, because they would still be receiving similar antibiotic treatments.

WHAT ARE THE BENEFITS OF PARTICIPATING IN THIS STUDY?

You may or may not benefit directly from participating in this study. Your participation may or may not help other people with blood stream infection in the future but by participating in this research, you may contribute to the knowledge and development of new treatment strategies for blood stream infections in the future.

CAN PARTICIPATION IN THIS STUDY END EARLY?

It is possible that the study investigators may stop the study, or your participation in it, at any time without your consent. If you are removed from this study, the investigator(s) will discuss the reasons with you.

You can also choose to end your participation at any time without providing a reason. If you choose to withdraw from the study, this will not have any effect on your current or future medical treatment or health care.

If you withdraw voluntarily from the study, you are encouraged to contact Dr. Nick Daneman on the contact details provided in the beginning of this document

WHAT ARE THE COSTS OF PARTICIPATING IN THIS STUDY?

Participation in this study will not involve any additional costs to you/your family member.

ARE STUDY PARTICIPANTS PAID TO PARTICIPATE IN THIS STUDY?

You will not be paid to participate in this study.

HOW WILL MY INFORMATION BE KEPT CONFIDENTIAL?

You have the right to have any information about you and your health that is collected, used or disclosed for this study to be handled in a confidential manner.

If you decide to participate in this study, the investigator(s) and study staff will look at your personal health information and collect only the information they need for this study. Personal health information" is health information about you that could identify you because it includes information such as your;

- name
- address
- telephone number
- date of birth
- new and existing medical records, or
- the types, dates and results of various tests and procedures.

You have the right to access, review and request changes to your personal health information. The following people may come to the hospital to look at your personal health information to check that the information collected for the study is correct and to make sure the study followed the required laws and guidelines:

- Representatives of the Ministry of Health and Long-Term Care Academic Health Sciences

- Representatives of the Sunnybrook Research Institute, Sunnybrook Health Sciences Centre or the Sunnybrook Research Ethics Board, because they oversee the ethical conduct of research studies at Sunnybrook

Access to your personal health information will take place under the supervision of the Principal Investigator. “Study data" is health information about you that is collected for the study, but that does not directly identify you. Any study data about you that is sent outside of the hospital will have a code and will not contain your name or address, or any information that directly identifies you. Study data that is sent outside of the hospital will be used for the research purposes explained in this consent form.

The investigator(s), study staff and the other people listed above will keep the information they see or receive about you confidential, to the extent permitted by applicable laws. Even though the risk of identifying you from the study data is very small, it can never be completely eliminated.

The Principal Investigator will keep any personal health information about you in a secure and confidential location for 10 years and then destroy it according to Sunnybrook policy. When the results of this study are published, your identity will not be disclosed.
You have the right to be informed of the results of this study once the entire study is complete. If you would like to be informed of the results of this study, please provide your name, address and telephone number to Asgar Rishu, at 416-480-6100-ext 88153 or Dr. Nick Daneman at 416-480-6100 x2791.

DO THE INVESTIGATORS HAVE ANY CONFLICTS OF INTEREST?
No- there are no conflicts of interest to declare related to this study.

COMMUNICATION WITH YOUR FAMILY DOCTOR
You may want to inform your family doctor of your participation in this study.

WHAT ARE THE RIGHTS OF PARTICIPANTS IN A RESEARCH STUDY?

- You have the right to receive all information that could help you make a decision about participating in this study.

- You also have the right to ask questions about this study and your rights as a research participant, and to have them answered to your satisfaction, before you make any decision.
- You have the right to ask questions and to receive answers throughout this study.
- Participating in this study is your choice (voluntary). You have the right to choose not to participate, or to stop participating in this study at any time without having to provide a reason.
- If you have any questions about this study you may contact the person in charge of this study (Principal Investigator, Dr. Nick Daneman, Department of Medicine Division of Infectious Diseases, contact: 416-480-6100 x2791, Pager: 7717).
- The Sunnybrook Research Ethics Board has reviewed this study. If you have questions about your rights as a research participant or any ethical issues related to this study that you wish to discuss with someone not directly involved with the study, you may call the **Chair of the Sunnybrook Research Ethics Board at (416) 480-6100 ext. 88144.**

Sunnybrook Health Sciences Centre
2075 Bayview Avenue,
Toronto, ON Canada M4N 3M5
t: 416.480.6100
www.sunnybrook.ca

DOCUMENTATION OF INFORMED CONSENT

You will be given a copy of this informed consent form after it has been signed and dated by you and the study staff.

Full Study Title: Bacteremia Antibiotic Length Actually Needed for Clinical Effectiveness (BALANCE): A Pilot Randomized Controlled Clinical Trial

Name of Participant: ____

Participant/Substitute decision-maker

By providing voluntary consent in writing, I confirm that:

- This research study has been fully explained to me and all of my questions answered to my satisfaction
- I understand the requirements of participating in this research study
- I have been informed of the risks and benefits, if any, of participating in this research study
- I have been informed of any alternatives to participating in this research study
- I have been informed of the rights of research participants
- I have read each page of this form
- I authorize access to my personal health information, medical record and research study data as explained in this form
- I have agreed, or agree to allow the person I am responsible for, to participate in this research study
- I understand that my family doctor may be informed of my participation in this research study

☐ I **agree** to allow my blood sample(s) to be collected for the study as described in this consent form.

☐ I **do not agree** to allow my blood sample(s) to be collected for the study as described in this consent form.

_________________________	_________________________	_________________________
Name of participant/Substitute decision-maker (print)	Signature	Date

Sunnybrook
HEALTH SCIENCES CENTRE

Sunnybrook Health Sciences Centre
2075 Bayview Avenue,
Toronto, ON Canada M4N 3M5
t: 416.480.6100
www.sunnybrook.ca

Assistance Declaration

Was the participant assisted during the consent process? ☐ Yes ☐ No

☐ The consent form was read to the participant/substitute decision-maker, and the person signing below attests that the study was accurately explained to, and apparently understood by, the participant/substitute decision-maker.

☐ The person signing below acted as a translator for the participant/substitute decision-maker during the consent process. He/she attests that they have accurately translated the information for the participant/substitute decision-maker, and believe that participant/substitute decision-maker has understood the information translated.

______________________________ ______________________________ ______________________

Name of Person Assisting (Print) Signature Date

Person obtaining consent

By signing this form, I confirm that:

- This study and its purpose has been explained to the participant named above
- All questions asked by the participant have been answered
- I will give a copy of this signed and dated document to the participant

______________________________ ______________________________ ______________________

Name of Person obtaining consent (print) Signature Date

Statement of Investigator

I acknowledge my responsibility for the care and well being of the above participant, to respect the rights and wishes of the participant as described in this informed consent document, and to conduct this study according to all applicable laws, regulations and guidelines relating to the ethical and legal conduct of research.

______________________________ ______________________________ ______________________

Name of Investigator (print) Signature Date
